# Epidemiology of trauma in the subarctic regions of the Nordic countries

**DOI:** 10.1186/s12873-021-00559-4

**Published:** 2022-01-11

**Authors:** Tine Steinvik, Lasse Raatiniemi, Brynjólfur Mogensen, Guðrún B. Steingrímsdóttir, Torfinn Beer, Anders Eriksson, Trond Dehli, Torben Wisborg, Håkon Kvåle Bakke

**Affiliations:** 1 Anaesthesia and Critical Care Research Group, University of Tromsø, The Arctic University of Norway, Tromsø, Norway; 2 Centre for prehospital emergency medicine, Oulu university hospital, Oulu, Finland; 3 Department of Anaesthesia and Intensive Care, Hammerfest Hospital, Finnmark Health Trust, Hammerfest, Norway; 4 University Hospital of Iceland Hringbraut 101, 101 Reykjavík, Iceland; 5 University of Iceland, Sæmundargata 4, 102 Reykjavík, Iceland; 6 Department of Emergency Medicine, Landspítali University Hospital, Fossvogur, 108 Reykjavík, Iceland; 7 Unit of Forensic Medicine, Department of Community Medicine and Rehabilitation, Umeå University, Umeå, Sweden; 8 The National Board of Forensic Medicine, Stockholm, Sweden; 9 Department of Gastrointestinal Surgery, University Hospital of North Norway, Tromsø, Norway; 10 Norwegian National Advisory Unit on Trauma, Division of Emergencies and Critical Care, Oslo University Hospital, Oslo, Norway; 11 Department of Anaesthesiology and Intensive Care, University Hospital of North Norway, Tromsø, Norway; 12 Trauma section, Department of Cardiothoracic and Vascular Surgery, University Hospital of North Norway, Tromsø, Norway; 13 Department of Health and Care Sciences, Faculty of Health Science, University of Tromsø, The Arctic University of Norway, Tromsø, Norway

**Keywords:** Trauma, Injury, Epidemiology, Rural

## Abstract

**Background:**

The northern regions of the Nordic countries have common challenges of sparsely populated areas, long distances, and an arctic climate. The aim of this study was to compare the cause and rate of fatal injuries in the northernmost area of the Nordic countries over a 5-year period.

**Methods:**

In this retrospective cohort, we used the Cause of Death Registries to collate all deaths from 2007 to 2011 due to an external cause of death. The study area was the three northernmost counties in Norway, the four northernmost counties in Finland and Sweden, and the whole of Iceland.

**Results:**

A total of 4308 deaths were included in the analysis. Low energy trauma comprised 24% of deaths and high energy trauma 76% of deaths. Northern Finland had the highest incidence of both high and low energy trauma deaths. Iceland had the lowest incidence of high and low energy trauma deaths. Iceland had the lowest prehospital share of deaths (74%) and the lowest incidence of injuries leading to death in a rural location. The incidence rates for high energy trauma death were 36.1/100000/year in Northern Finland, 15.6/100000/year in Iceland, 27.0/100000/year in Northern Norway, and 23.0/100000/year in Northern Sweden.

**Conclusion:**

We found unexpected differences in the epidemiology of trauma death between the countries. The differences suggest that a comparison of the trauma care systems and preventive strategies in the four countries is required.

## Introduction

Annually, injuries kill 5.8 million people worldwide. Injuries are one of the top three leading causes of death for people aged 15 to 49 years, resulting in a significant impact on life years lost [1]. However, an increase in knowledge, preventive measures, efficiency and technology in emergency medicine, and implementation of trauma systems has contributed to increasing survival from serious injuries over the past few years [1, 2].

The burden of injury is not evenly distributed. Rural areas, with sparse population and long distances, have higher death rates from injuries than urban [3–6]. Urban-rural differences has been attributed to longer response and transport distances, behavioural differences and differences in socio-economic status [4].

The northern subarctic areas of the Nordic countries are, in a European context, characterised by large areas with low population density. Epidemiological findings from other rural areas are not necessarily applicable or transferable. The majority of trauma research in rural areas is from the USA, Canada, and Australia, countries with areas that have considerably longer transport distances, but also other societal differences. Differences in climate, politics, and demographics, necessitate local studies.

The northernmost areas of the Nordic countries have common challenges to trauma care, with regards to distance, rurality, and climate. Although some structural societal differences between the Nordic countries are likely as well, they are relatively homogenous, with small differences in socioeconomic status on a national level. Therefore, we believe that a head-to-head comparison of trauma epidemiology between these northern regions, may disclose areas where these Nordic neighbours can learn from one another. While the differences in overall epidemiology must be expected to be small, any differences may point to areas where the otherwise similar regions can look to the others for ways to improvement. In addition, though formalised trauma systems have been implemented in the Nordic countries, insight into the local epidemiology of trauma may help adjust these systems to local needs [7].

The aims of the present study were to describe and compare the rates of fatal injuries in the northern regions of the Nordic countries between the countries, and to describe and compare the injury mechanisms and places of death.

## Materials and methods

### Study design

A registry based retrospective cohort study.

### Study area

The study area comprised the northernmost university hospital and its catchment area, for the Nordic countries. Thus, the study area included Iceland in its entirety, the three northernmost counties in Norway, and the four northernmost counties in both Finland and Sweden. The area covers approximately 730,000 km^2^ and had a population of nearly 2.4 million in 2011.

The four northernmost counties in Finland are Kainuu, Lapland, Middle Ostrobothnia, and Northern Ostrobothnia. Together these counties cover approximately 166,000 km^2^, 731,000 inhabitants (14% of Finland’s population), and had one trauma centre (analogue to a level II hospital according to the American College of Surgeon’s Trauma Centre Classification System, with all relevant specialties available, but not fulfilling the required volume of a level I hospital) and four local hospitals with trauma function (analogue to level III hospitals, with capability to initially manage the majority of injured patients but with transfer agreements with a higher level trauma centre) in 2011 [8]. Iceland covers approximately 103,000 km^2^ and 418,000 inhabitants, and had one Level II and one Level III hospital [9]. The three northernmost counties in Norway were Finnmark, Nordland and Troms, which cover approximately 177,000 km^2^, 470,000 inhabitants (10% of Norway’s population), and had one Level II and nine Level III hospitals in 2011 [10]. The four northernmost counties in Sweden are, Norrbotten, Västerbotten, Jämtland and Västernorrland. These counties cover approximately 225,000 km^2^, 877,000 inhabitants (9% of Sweden’s population), and had one Level II and 11 Level III hospitals in 2011 [11].

### Inclusion and exclusion criteria

**Fig. 1:**
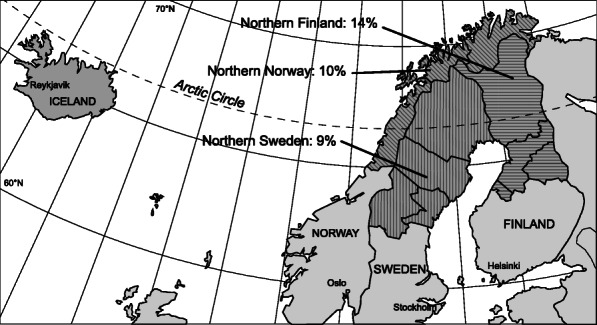
Map of the study area. Percentages of the population under study in the respective countries are specified where applicable. The Map was created using Adobe Illustrator CC 2020 (24.0) (), by Rod Wolstenholme, UiT

We identified from the national Cause of Death Registries and recorded all individuals who died from external causes (ICD-10 codes V01-Y98) in the northern regions of the Scandinavian Peninsula, and Iceland (Fig. 1) for the 5-year period from 2007 through 2011 [12].

**Fig. 2:**
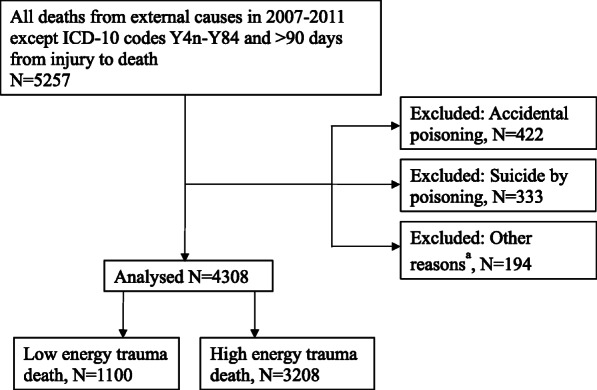
Flowchart of case inclusion and exclusion. High energy trauma deaths include traumatic suicides. ^a^Other reasons include cases with too much information missing to register in the database, no hospital record found, no personal identification number found, ICD-code(s) not matching inclusion criteria, and incompatible differences in the method of gathering LE data in Sweden (*n* = 60)

We excluded all individuals with iatrogenic injuries (ICD-10 codes Y40-Y84) and all deaths occurring more than 90 days after injury. We also excluded cases in which key information was lacking, such as cases with an injury in an undetermined municipality. We excluded all accidental poisonings, except carbon monoxide poisoning where fire was the mechanism (Fig. 2). In addition, cases with too much information missing to register in the database, no hospital record found, no personal identification number found, were also excluded. These constituted 194 patients: 50 patients from Norway, 61 from Iceland, 23 from Finland, in addition to 60 patients with low energy trauma from Sweden.

### Data collection and analysis

We gathered data from the Cause of Death Registries (CDRs) in Finland, Iceland, and Norway, and from the Unit of Forensic Medicine in Umeå of the National Board of Forensic Medicine in Sweden. The method for data gathering in Sweden omitted many cases, particularly low energy trauma, because deaths due to ground level falls are not routinely autopsied. Because of this, Swedish data were exempt from the low energy comparison. Researchers from each country gathered their own data. We anonymised the data by assigning each case an identifier and removed personal identification numbers, keeping the key for reversal and the data separate. The anonymised data were transferred to a common database for analysis. The data could still be identified indirectly as single data points, but not when summarised as rates and proportions.

Age, sex, date of death, municipality of residence, municipality of injury, ICD-10 codes for the cause of death, and how the cause of death was determined were collected from the CDRs and Unit of Forensic Medicine [12]. The intention, mechanism, and dominating type of injury and site of death, or place of death if not on site, were obtained from the CDRs, hospital records, and autopsy reports. In Finland and Sweden, we used insurance records and police reports, respectively, to supplement the information required by the study.

We analysed all high energy trauma deaths (including trauma suicides), and low energy trauma deaths separately. The proportions given below are the proportions of collated deaths. Incidence rates were calculated using the 2011 population size as denominator, and municipality of injury as numerator. Population size was obtained from the national statistics provider from each country (Statistics Norway, Statistics Finland, StatIce, and Statistics Sweden) [8–11]. The OECD standard population of 1980 was used for age and sex standardisation [13]. All rates are given in number of deaths/100000/year (95% confidence interval [CI]).

### Definitions

We categorised the municipalities into four levels of rurality based on the municipality’s population density. Level 1 (most rural) was defined as < 18.15 inhabitants/km^2^, level 2 as 18.15–76.9 inhabitants/km^2^, level 3 as 77.0–442.7 inhabitants/km^2^, and level 4 (most urban) as > 442.7 inhabitants/km^2^ [14]. We assigned an “urban” (level 3) category to the municipalities with a university hospital, even if this municipality had a lower category based on population density, and levels 3 and 4 were grouped together as “urban” during the analysis.

We divided the manner of death into the main categories of unintentional (accidental), intentional by self (suicidal), intentional by other (homicidal), other, and unknown. We defined low energy trauma as ground level falls, whereas the remaining trauma cases were regarded as high energy trauma.

We used the American College of Surgeon’s Trauma Centre Classification System to define university hospitals with a neurosurgery department as trauma centres (Level II), and smaller hospitals equipped to deal with traumatic injuries as local hospitals with trauma function (Level III) [15]. If the hospital did not admit trauma patients according to the national trauma system, the hospital was left out of the registration.

### Ethics

According to Swedish law, register studies of deceased individuals do not require ethical approval. The study was approved by ethics committees in each of the other countries: Regional Ethics Committee, Finland (98/2013); The National Bioethics Committee, Vísindasiðanefnd, Iceland (VSNb201410013/03.11); The Norwegian Ethics Board (REK) (No. 2013/1470/REK Nord); and Patient Data Security Officer at University Hospital of Northern Norway (2014/0418).

### Statistical analysis

Statistical analyses were performed using SPSS (IBM SPSS Statistics for Windows, Version 25.0, Armonk, NY, USA). We present the results using medians with interquartile range (IQR), percentages with 95% CIs, and crude incidence rates of 100,000/year with 95% CIs. We used 95% CIs to determine if there were any differences between the countries and between the crude rates of subgroups.

## Results

We collated a total of 5190 cases, with 4641 cases remaining after exclusion (Fig. 2). Low energy trauma deaths accounted for 1100 cases, whereas 3208 cases were high energy trauma deaths.

Low energy (LE) trauma comprised 24% of all included cases. Victims of low energy trauma were older (median age 84 years), and with a more equal sex distribution (male sex in 51% of cases) compared to victims of high energy trauma, and death mostly occurred in-hospital (in-hospital death in 88.5% of cases). Comparison of LE trauma deaths between the study areas can be found in Table 1. Iceland had a significantly lower incidence of LE trauma compared to the other areas.

**Table 1:** Low energy (LE) trauma deaths by study region

	Northern Finland	Iceland	Northern Norway	Total study area
Number of LE trauma deaths registered	610	104	386	1100
Incidence of LE trauma deaths per 100,000 per year (95% CI)	16.7 (15.3–18.0)	6.5^a^ (5.3–7.8)	16.5 (14.8–18.1)	14.5 (13.6–15.4)
Autopsy proportion (95% CI)	419/610^a^ 68.7% (65.0–72.4)	14/104^a^ 13.5% (6.9–20.0)	123/386^a^ 31.9% (27.2–36.5)	556/1100 50.5% (47.6–53.5)
Prehospital deaths (95% CI)	94/610^a^ 15.4% (12.5–18.3)	11/104 10.6% (4.7–16.5)	22/386^a^ 5.7% (3.4–8.0)	127/1100 11.5% (9.7–13.4)
Median age, years (IQR)	82.0 (69–88)	86.0 (79–90)	87.0 (82–91)	84.0 (76–89)
Male sex (95% CI)	348/610^a^ 57.0% (53.1–61.0)	51/104 49.0% (39.4–58.6)	162/386^a^ 42.0% (37.0–46.9)	561/1100 51.0% (48.0–54.0)
Rural injury municipality proportion (95% CI)	283/610^a^ 46.4% (42.4–50.4)	6/104^a^ 5.8% (1.3–10.3)	264/386^a^ 68.4% (63.8–73.0)	553/1100 50.3% (47.3–53.2)
Incidence of LE trauma death after injury in rural municipality (95% CI)	16.4 14.4–18.3	2.0^a^ 0.4–3.6	17.7 15.6–19.8	15.7 14.4–17.7
Incidence of LE trauma death after injury in semi-urban municipality (95% CI)	15.0 12.3–17.8	9.1 1.1–17.1	17.1 13.5–20.7	15.6 13.5–17.7
Incidence of LE trauma death after injury in urban municipality (95% CI)	18.4^a^ 15.9–20.8	7.5 6.0–9.1	10.3 6.3–13.7	12.4 11.1–13.7

^a^Significant difference (from one or all). Limited data were available on low energy trauma deaths from Northern Sweden and were not included in the comparison

High energy (HE) trauma comprised 76% of all included cases. Blunt force trauma was the most prevalent type of injury, followed by asphyxia and penetrating injuries. Most HE trauma deaths occurred in the prehospital phase in all study areas. Median age was 50 years, and males constituted 80% of HE deaths. A comparison of HE trauma deaths between the study areas can be found in Table 2. Finland had the highest incidence rate of HE trauma, followed by Norway, Sweden, and Iceland. Age and sex standardisation (Table 3) did change the absolute rates, but not their internal order among the countries.

**Table 2:** High energy (HE) trauma deaths by study region

	Dominating type of external cause	Northern Finland	Iceland	Northern Norway	Northern Sweden	Total
Number of HE trauma deaths registered		1321	249	633	1006	3208
Incidence in 100,000 per year (95% CI)		36.1^a^ (34.2–38.1)	15.6^a^ (13.7–17.6)	27.0^a^ (24.9–29.1)	23.0^a^ (21.5–24.4)	26.8 (25.9–27.7)
Autopsy proportion (95% CI)		1314/1321^a^ 99.5% (99.1–99.9)	228/249^a^ 91.6% (88.1–95.0)	232/633^a^ 36.7% (32.9–40.4)	^b^	1774/2203 80.5% (78.9–82.2)
Prehospital deaths (95% CI)		1121/1321^a^ 84.9% (82.9–86.8)	183/249^a^ 73.5% (68.0–79.0)	517/633^a^ 81.7% (78.7–84.7)	921/1006^a^ 91.6% (89.8–93.3)	2742/3208 85.5% (84.3–86.7)
Median age, years (IQR)		51.0 (33–64)	45.0 (31–60)	49.0 (31–66)	52.0 (34–66)	50.0 (33–65)
Male sex (95% CI)		1062/1321 80.4% (78.3–82.5)	203/249 81.5% (76.7–86.3)	486/633 76.8% (73.5–80.1)	824/1006 81.9% (79.5–84.3)	2575/3208 80.3% (78.9–81.6)
Rural injury municipality proportion (95% CI)		577/1321^a^ 43.7% (41.0–46.4)	72/249^a^ 28.9% (23.3–34.5)	456/633^a^ 72.0% (68.5–75.5)	643/1006^a^ 63.9% (60.9–66.9)	1747/3208 54.5% (52.7–56.2)
Incidence of HE trauma death after injury in rural municipality (95% CI)		33.3^a^ 30.6–36.1	23.8^a^ 18.3–29.3	30.6 27.8–33.4	26.4^a^ 24.3–28.4	29.3 27.9–30.7
Incidence of HE trauma death after injury in semi-urban municipality (95% CI)		33.0^a^ 29.0–37.0	7.3* 0.1–14.4	19.6 15.8–23.5	21.1 18.6–23.5	24.0 22.2–25.8
Incidence of HE trauma death after injury in urban municipality (95% CI)		42.6^a^ 38.8–46.4	14.0 11.9–16.1	22.6^a^ 17.5–27.6	13.1 10.1–16.0	24.6 22.9–26.3
Incidence per 100,000 per year (95% CI)	Blunt force trauma		5.8^a^ (4.7–7.0)	9.3 (8.1–10.5)	8.1 (7.2–8.9)	9.0 (8.4–9.5)
Incidence per 100,000 per year (95% CI)	Penetrating trauma	6.7^a^ (5.9–7.6)	2.0 (1.3–2.7)	2.9 (2.2–3.5)	0.9^a^ (0.6–1.2)	3.2 (2.9–3.5)
Incidence per 100,000 per year (95% CI)	Hypothermia	2.3^a^ (1.8–2.8)	0.4 (0.1–0.8)	1.1 (0.6–1.5)	1.0 (0.7–1.3)	1.3 (1.1–1.5)
Incidence per 100,000 per year (95% CI)	Asphyxia	8.1^a^ (7.2–9.0)	5.0^a^ (3.9–6.1)	7.0 (5.9–8.1)	6.9 (6.1–7.6)	7.0 (6.5–7.5)
Incidence per 100,000 per year (95% CI)	Submersion	5.1^a^ (4.4–5.9)	1.5^a^ (0.9–2.1)	3.9 (3.1–4.7)	3.1 (2.6–3.6)	3.7 (3.3–4.0)

^a^Significant difference, no overlap of 95% confidence intervals

^b^Not applicable. Swedish data contained only cases that underwent autopsy, and the exact proportion could not be established

**Table 3:** Incidence rates of High Energy (HE) and Low Energy (LE) trauma deaths adjusted for age and sex

	Northern Finland	Iceland	Northern Norway	Northern Sweden	Total
**HE trauma**					
Incidence per 100,000 per year (95% CI)	32.1^a^ (30.3–33.8)	14.6^a^ (12.8–16.4)	25.0^a^ (23.0–26.9)	20.3^a^ (19.1–21.6)	24.0 (23.2–24.9)
**LE trauma**					
Incidence per 100,000 per year (95% CI)	10.8 (9.9–11.6)	5.7^a^ (4.6–6.8)	11.0 (9.9–12.1)	–	7.1 (6.7–7.5)

^a^Significant difference (from one or all)

Suicide was the most common manner of death, comprising 40% of all high energy trauma deaths, followed by accidental deaths in traffic. A comparison of mechanism of injury for high energy trauma between the study areas can be found in Table 4. The distribution was fairly similar internally in each area, with Finland significantly higher for several mechanisms. The difference between areas was greatest for suicide where Finland had an incidence rate of 15.6 per 100.000 compared to 9.3 per 100.000 in Northern Sweden, which had the second highest incidence rate for suicide.

**Table 4:** Annual incidence rate per 100,000 (95% CI) of the manner and mechanism of death intentions and mechanisms of injury in high energy trauma, including traumatic suicides

Manner of death	Mechanism of death	Subgroup	Northern Finland	Iceland	Northern Norway	Northern Sweden	Total
Accident			16.9^a^ (15.5–18.2)	7.2^a^ (5.9–8.5)	15.6 (14.0–17.2)	12.3^a^ (11.3–13.4)	13.7 (13.0–14.3)
	Road transport^†^		6.9^a^ (6.0–7.7)	3.4^a^ (2.5–4.3)	6.6 (5.5–7.6)	5.1^a^ (4.4–5.7)	6.2 (5.8–6.7)
		Car, pickup, heavy transport, bus, etc.	3.8 (3.1–4.4)	2.3^a^ (1.6–3.1)	4.4^a^ (3.6–5.3)	3.5 (3.0–4.1)	3.8 (3.5–4.2)
		Pedestrian	0.9 (0.6–1.2)	0.5 (0.2–0.9)	0.5 (0.2–0.7)	0.4 (0.2–0.6)	0.8 (0.7–1.0)
		Motorcycle	0.5 (0.2–0.7)	0.5 (0.2–0.9)	0.6 (0.3–1.0)	0.8 (0.5–1.0)	0.6 (0.5–0.8)
		Bicycle	0.7^a^ (0.4–0.9)	0.0^a^ (0.0–0.0)	0.2 (0.0–0.4)	0.1^a^ (0.0–0.2)	0.3 (0.2–0.4)
		ATV/ Snowmobile	1.0 (0.7–1.3)	0.0^a^ (0.0–0.0)	0.4^a^ (0.2–0.7)	1.1^a^ (0.8–1.4)	0.8 (0.6–1.0)
		Other traffic	1.1*^a^ (0.7–1.4)	0.1^a^ (0.0–0.2)	0.8^a^ (0.4–1.2)	0.3 (0.1–0.4)	0.7 (0.5–0.8)
	High fall		2.1^a^ (1.6–2.5)	1.7 (1.1–2.3)	2.4^a^ (1.8–3.1)	1.1^a^ (0.8–1.4)	1.7 (1.5–2.0)
	Submersion		3.8 (3.1–4.4)	1.5^a^ (0.9–2.1)	2.9 (2.2–3.6)	2.8 (2.3–3.3)	3.0 (2.6–3.3)
	Fire		1.5 (1.1–1.9)	0.3^a^ (0.0–0.6)	2.0 (1.4–2.5)	1.1 (0.8–1.5)	1.4 (1.2–1.6)
	Hypothermia		2.2^a^ (1.7–2.7)	0.4 (0.1–0.8)	0.9 (0.5–1.3)	1.0 (0.7–1.3)	1.3 (1.1–1.5)
	Other		8.8 (7.8–9.7)	5.3^a^(4.1–6.4)	7.8^a^(6.6–8.9)	6.2^a^(5.5–7.0)	7.2 (6.7–7.7)
Homicide			2.3^a^(1.8–2.8)	0.4 (0.1–0.8)	0.6 (0.3–0.9)	0.6 (0.3–0.8)	1.1 (0.9–1.3)
	Knife/stabbing		0.9^a^(0.6–1.2)	0.1^a^(0.0–0.2)	0.3 (0.1–0.6)	0.5^a^(0.3–0.7)	0.5 (0.4–0.7)
	Shooting		0.7^a^(0.4–1.0)	0.1 (0.0–0.3)	0.1 (0.0–0.2)	0.0 (0.0–0.0)	0.2 (0.2–0.3)
	Blunt force trauma		0.3 (0.1–0.5)	0.2 (0.0–0.4)	0.1 (0.0–0.2)	0.0 (0.0–0.1)	0.2 (0.1–0.2)
	Other		0.3 (0.1–0.5)	0.1 (0.0–0.2)	0.1 (0.0–0.2)	0.1 (0.0–0.1)	0.1 (0.0–0.1)
Suicide			15.6^a^(14.4–16.8)	7.0^a^(5.7–8.3)	8.8 (7.6–10.0)	9.3^a^(8.4–10.2)	10.8 (10.2–11.4)
	Hanging		6.5^a^(5.7–7.3)	3.6^a^(2.7–4.6)	5.1 (4.2–6.0)	5.5^a^(4.8–6.2)	5.5 (5.0–5.9)
	Shooting		5.0^a^(4.2–5.7)	1.4 (0.9–2.0)	1.9 (1.3–2.4)	2.3 (1.8–2.7)	2.9 (2.6–3.2)
	Drowning		1.3^a^(1.0–1.7)	0.4 (0.1–0.7)	0.6 (0.3–0.9)	0.5 (0.3–0.7)	0.8 (0.6–0.9)
	Traffic		1.4^a^(1.0–1.8)	0.6 (0.2–0.9)	0.2 (0.0–0.4)	0.3 (0.1–0.4)	0.6 (0.5–0.8)
	Other external cause of death		1.4^a^(1.0–1.8)	1.0 (0.5–1.5)	1.1 (0.7–1.5)	0.7 (0.5–1.0)	1.0 (0.9–1.2)

^a^Significant difference (from one or all), no overlap of 95% confidence intervals

^b^All transport accidents on air, sea, and land

## Discussion

The aims of the present study were to describe and compare the rates of fatal injuries in the northern regions of the Nordic countries, and to describe and compare the injury mechanisms and places of death. The areas differed in the overall incidence rate of high energy trauma, with some accompanying differences in the manner and external cause of death. Northern Finland had the highest incidence, primarily driven by a high suicide rate. Deaths from high energy trauma were mostly prehospital (74–92%) in all areas; Iceland had the lowest share of prehospital high energy trauma deaths but was also the area with the lowest incidence of rural deaths. Autopsy rates were high (92–100%), except in Northern Norway (36%). The pattern of low energy trauma was more homogenous between the areas. Low energy trauma deaths occurred in older individuals, predominantly during primary admission or after being discharged, and with a more equal balance between the sexes than high energy trauma deaths.

The northern part of the Scandinavian peninsula and Iceland face similar challenges in trauma care, with sparsely populated areas, long transport distances and prehospital times in a European perspective. Differences in incidence of trauma fatalities between the northern areas, and the denser southern areas have been shown in previous studies [6, 16, 17]. The present study had injury fatality rates that exceeded those reported in studies from the southern parts of the Nordic countries [6, 18]. Despite these similar challenges for the northern areas of the Nordic countries, the present study reveals that there are marked differences between the study areas.

Iceland had a lower incidence of high energy trauma death, and a lower share of prehospital deaths. Rural areas have a higher rate of fatal injuries than urban areas, and typically a higher share of prehospital deaths [4, 19, 20]. A more urban settlement pattern in Iceland could explain Iceland’s deviant profile. Interestingly, Iceland also had a lower incidence of fatalities caused by low energy trauma, where previous studies have shown less tendency to urban-rural disparity, or even increased urban incidence rates [5, 21, 22].

There were also some differences between the other areas; Northern Finland, Northern Sweden, and Northern Norway. These areas have more similar settlement patterns, with the majority of the population living in non-urban areas, yet Northern Finland had a significantly higher incidence rate of fatal trauma, both for high energy and low energy trauma deaths [10, 11, 23]. Their incidence rates were comparatively high for most injury mechanisms overall, but particularly for suicides and homicides. The suicide rate was the main driver for the overall difference in high energy trauma death incidence between northern Finland and its neighbours.

When considering suicide, it is important to keep in mind that poisoning is a common method [24]. In trauma studies where poisonings are excluded a perceived difference in suicide rates may reflect a difference in the choice of method, and not a real difference in suicide rates. Indeed, if poisonings are considered when comparing suicide rates between the study areas of this study, only Northern Finland has a significantly different suicide rate compared to the other areas (unpublished data). Therefore, this may be a point of attack for reduction of the Finnish excessive incidence of fatal trauma, in the form of preventive strategies.

In addition to a markedly elevated suicide rate compared to the other study areas, northern Finland also had the highest incidence rate for several other mechanisms. Lower socioeconomic status and comorbidity are known risk factors for trauma related death, as well as high ethanol consumption or an unfavourable drinking pattern [4, 25, 26]. As Finland has a comparatively similar settlement pattern as its neighbours, these would be areas for further study to find explanations for the higher mortality rates. Another area to look into would be the structure and capacities of the trauma health care systems in the study areas.

Autopsy rates was another area of considerable difference. Northern Norway had a markedly lower autopsy rate compared to the other areas. Epidemiological data from a population with a higher autopsy rate are likely more reliable, and autopsies are useful in determining the correct cause of death and to assess whether deaths are preventable. In Finland and Iceland, an autopsy was performed in almost all deaths, which suggests that this also should be feasible in Norway and is an area for improvement.

Considering the northern parts of the Nordic countries as one area, the overall age and sex adjusted annual rate of injury-related deaths in the present study was 31.1/100000 (24.0 for high energy trauma deaths and 7.1 for low energy trauma deaths). This is well below the mortality rates Mack et al. has reported for the USA, more in line with, and slightly lower than their findings from Canada and Australia overall [27]. However, the incidence rate was not as high as the more rural areas of both Australia and Canada [28, 29].

### Limitations

This study has several limitations. First, it is retrospective and includes only fatalities; therefore, we could not assess survivability, which would be of great interest to compare between the regions. The Swedish data were collated from the Unit of Forensic Medicine and not from the CDR. This constitutes a problem for the analysis of Swedish low energy cases, and these were exempt from comparison to the other areas. Autopsy rates for high energy trauma in Sweden have been reported to be quite high, and the inclusion through the Unit of Forensic Medicine should capture most cases [30, 31]. Even so, the Swedish high energy trauma deaths should be interpreted with care. The varying autopsy rates aggravate a comparison of injury severity scores, which could be useful in analysing differences in mortality rates. This also makes it impossible to accurately describe the mechanism of death beyond the external cause of death.

## Conclusion

The northern regions of the Nordic countries have an overall rural injury pattern with relatively high rates of trauma deaths and predominantly prehospital fatalities. Northern Finland has a high injury death rate compared to its neighbours, a difference that is largely driven by a high rate of suicide. Iceland has a comparatively lower injury death rate. Given the differences in regions that would be expected to be relatively similar, further studies should investigate social differences and different approaches to health care.

## Data Availability

The datasets used and/or analysed during the current study are available from the corresponding author on reasonable request.

## Abbreviations

CDR: Cause of Death Registry
LE: Low energy
HE: High energy
CI: Confidence interval
